# Supplementary material for: Treatment of Ebola Virus Disease: From Serotherapy to the Use of Monoclonal Antibodies
Source: Antibodies (Basel). 2025 Mar 5;14(1):22. doi: 10.3390/antib14010022 (PMC11939263; doi:10.3390/antib14010022)
Supplement: Supplementary file 1 [file antibodies-14-00022-s001.zip › antibodies-3387567-supplementary.pdf]

Supplementary Materials

Table S1. Antibodies interacting with EBOV GP

Name	Production method	Source	Interaction region, epitope	Virus neutralization	Ref.
6D8-1-2, c6D8	hybridomas	mouse	388HNTP**VYKLDISEA**T QVE406, GP1, MLD	–	[41,42]
13F6-1-2 h-13F6	hybridomas	mouse	400ATQ**V**E**QHHRRTD**N DSTA418, GP1, MLD	–	
6D3-1-1	hybridomas	mouse	sGP	–	
13C6-1-1, c13C6	hybridomas	mouse	266SNT**T**G**K**LI275, GP1, sGP, GC	In the presence of complement IC50 > 1.0 mg/ml	
14G7	hybridomas	mouse	GKLGLITN**TIAGV**AGLI	No	
KZ52, KS56	phage display	human	GP1–GP2 interface, C511,N550, D552, G553, C556	$PRNT_{80}$ = 0.625 μg/ml, IC50 (lenti EBOV GP) = 0.06 μg/ml, IC90 (lenti EBOV GP) = 17.21 μg/ml (testing using pseudoviruses)	[35,36,38,41,47,54,73,110]
J3P-K11 (JP3K11)	phage display	monkey	GP1 and GP2	90% neutralization, 10 μg/ml	[38,115]
J3P-K9			unknown	–	
J3P-K14			NP	–	
133/3.16 (ch133)	hybridomas	mouse	GP2, 549 aa	IC50 (VSV EBOV GP) = 2.3 μg/ml, IC50 (EBOV) = 3.2 μg/ml (ch133, 3.2 μg/ml)	[46,47]
226/8.1 (ch226)			GP1, 134, 194, 199 aa	IC50 (VSV EBOV GP) = 0.6 μg/ml, IC50 (EBOV) = 0.8 μg/ml (ch226, 3.2 μg/ml)	
16F6	hybridomas	mouse	GP1–GP2 interface	90% neutralization, 20 nM, SUDV virus	[41,54,55,110]
E10	secondary phage library		GP1–GP2 interface	90% neutralization, 20 nM, SUDV virus, without complement	[55]
F4			GP1–GP2 interface	90% neutralization, 20 nM, SUDV virus, without complement	
1H3, c1H3	hybridomas	mouse	GP1, sGP, GC, 272LI**WK**VN**P**E281	1/200 50%, plaque formation inhibition test	[41,43,49,51,52]
2G4, c2G4			GP2, C511, N550, G553, C556	IC50 (VSV EBOV GP) 0.139 μg/ml	

4G7, c4G7			GP1 and GP2, C511, D552, C556	IC50 (VSV EBOV GP) 0.135 μg/ml	
m16G8	hybridomas	mouse	GP2		[77]
m8C4			GC	IC50 (EBOV) = 1.5 μg/ml, IC50 (SUDV) = 0.75 μg/ml	
m17C6			unknown		
m4B8			GP1		
m21D10			RBS, SATKRWGFRS		
S9	hybridomas	mouse	292TKKNLTRKIRSEELSC 308, GC	90% neutralization of 4 μg/ml EBOV	[74]
#3327	hybridomas	mouse	GP2, 526**IG**L530	–	[76]
ADI-15731	sorting	human	GC	$PRNT_{80}$ = 1.21 μg/ml	[57]
ADI-15734	sorting	human	GP1–GP2 interface	$PRNT_{80}$ = 0.28 μg/ml	
ADI-15758	sorting	human	HR2, GP base	$PRNT_{80}$ = 0.12 μg/ml	
ADI-15959	sorting	human	unknown	$PRNT_{80}$ = 1.51 μg/ml	
scFv-15999	protein engineering	human	GP base	IC50 (VSV EBOV GP) = 100 nM	[116]
scFv-15742	protein engineering	human	GP1–GP2 interface	IC50 (VSV EBOV GP) = 15 nM	
M318	phage library	mouse	GP1	IC50 (lenti EBOV) = 0.018 μg/ml, ADCC EC50 (lenti EBOV) = 0.095 μg/ml	[59]
M401	phage library	mouse	GP1	ADCC EC50 (lenti EBOV) = 0.087 μg/ml	
M501	phage library	mouse	GP1	IC50 (lenti EBOV) = 0.043 μg/ml, ADCC EC50 (lenti EBOV) = 0.097 μg/ml	
M001	phage library	mouse	GP1	-	
BDBV270	Immortalization of B cells	human	GC, W275	IC50 (BDBV) = 0.182 μg/ml	[84]
BDBV340	Immortalization of B cells	human	GP2	IC50 (BDBV) = 1.650 μg/ml	
mAb100	Immortalization of B cells	human	GP1–GP2 interface	IC50 (lenti EBOV GP) = 0.06 μg/ml, IC90 (lenti EBOV GP) = 0.61 μg/ml	[63]

mAb114	Immortalization of B cells	human	GC, GP1 core	IC50 (lenti EBOV GP) = 0.09 µg/ml, IC90 (lenti EBOV GP) = 0.71 µg/ml	
Q206	sorting	monkey	GC, GP1 core, T144	IC50 (lenti EBOV GP) = 0.36 µg/ml, IC90 (lenti EBOV GP) = 1.49 µg/ml, IC50 (EBOV) = 5.16 µg/ml	[60]
Q314	sorting	monkey	GC, E231, W275	IC50 (lenti EBOV GP) = 0.78 µg/ml, IC90 (lenti EBOV GP) = 6.77 µg/ml, IC50 (EBOV) = 38.9 µg/ml	
Q411	sorting	monkey	GC, GP1 core, T144	IC50 (lenti EBOV GP) = 0.43 µg/ml, IC90 (lenti EBOV GP) = 2.23 µg/ml, IC50 (EBOV) = 15.24 µg/ml, IC90 (lenti EBOV GP) = 61.65 µg/ml	
rEBOV-520	sorting	human	GP base	IC50 (Mouse-adapted EBOV Mayinga) = 2.7 µg/ml	[61,62]
rEBOV-548	sorting	human	GC	IC50 (Mouse-adapted EBOV Mayinga) = 11.8 µg/ml, IC90 (Jurkat-EBOV GP) = 41.7 µg/ml	
REGN 3470	sorting	VelocImmune mice	GC	IC50 (VSV EBOV GP) 0.27 nmol/L	[64,67,109]
REGN 3471	sorting	VelocImmune mice	GP1	IC50 (VSV EBOV GP) 0.39 nmol/L	
REGN 3479	sorting	VelocImmune mice	GP base and GP1–GP2 interface	IC50 (VSV EBOV GP) 0.17 nmol/L	
Cross-neutralizing					
M4	hybridomas	mouse	unknown	90% neutralization of 0.6 µg/ml MARV (Angola, Musoke, Ozolin, Ravn)	[74]
BDBV43	Immortalization of B cells	human	GC	IC50 (BDBV) = 139 ng/ml	[84]
BDBV324	Immortalization of B cells	human	GC, W275 and L273	IC50 (BDBV) = 10 ng/ml	
MR72	Immortalization of B cells	human	RBS, N129	Neutralization of EBOV, SUDV, RESTV, BDBV after treatment with cathepsin	[64,88]
FVM04	yeast display	monkey	RBS, GP1, K115, D117 and G118	IC50 (VSV EBOV GP) = 3,4 µg/ml, IC50 (VSV SUDV GP) = 4,3 µg/ml	[78,79]
FVM02p	yeast display	monkey	525AIGLAWIPYF536		
CA45	sorting	monkey	GP1–GP2 interface, R64, Y517, G546, N550	IC50 (VSV EBOV GP) = 4.4 nM, IC50 (VSV SUDV GP) = 12.0 nM, IC50 (VSV BDBV GP) = 0.9 nM, IC50 (EBOV) = 28.3 nM, IC50 (SUDV) = 78.2 nM, IC50 (BDBV) = 5.5 nM	[81]

m8C4	hybridomas	mouse	unknown	IC50 (EBOV) = 1.5 μg/ml, IC50 (SUDV) = 0.75 μg/ml	[77]
6D6	hybridomas	mouse	GP2, IFL, G529, L530	IC50 (VSV EBOV GP1976) = 0.05 μg/ml, IC50 (VSV EBOV GP2014) = 0,12 μg/ml, IC50 (VSV SUDV) = 0.19 μg/ml, IC50 (VSV TAFV) = 0.33 μg/ml, IC50 (VSV BDBV) = 0.24 μg/ml, IC50 (VSV RESTV) = 0.62 μg/ml	[83]
scKZ52-F4 LCN	protein engineering	mouse	GP1–GP2 interface	IC50 (VSV EBOV GP) = 3.8 nM, IC50 (VSV SUDV GP) = 12.0 nM, IC50 (EBOV) = 1.1 nM, IC50 (SUDV) = 1.0 nM	[56]
scKZ52-F4HCC	protein engineering		GP1–GP2 interface	IC50 (VSV EBOV GP) = 6.4 nM, IC50 (VSV SUDV GP) = 4.7 nM, IC50 (EBOV) = 0.8 nM, IC50 (SUDV) = 0.2 nM	
scKZ52-E10 LCN	protein engineering		GP1–GP2 interface	IC50 (VSV EBOV GP) = 3.3 nM, IC50 (VSV SUDV GP) = 20 nM, IC50 (EBOV) = 0.7 nM, IC50 (SUDV) = 1.5 nM	
scKZ52-E10HCC	protein engineering		GP1–GP2 interface	IC50 (VSV EBOV GP) = 1.5 nM, IC50 (VSV SUDV GP) = 3.8 nM, IC50 (EBOV) = 0.5 nM, IC50 (SUDV) = 0.2 nM	
FVM09-548	protein engineering	mouse	GC, 283GEWAF289, NPC	IC50 (VSV EBOV GP) = 2.9 nM, IC50 (VSV SUDV) = 60 nM, IC50 (VSV TAFV) = 3.0 nM, IC50 (VSV BDBV) = 1.3 nM, IC50 (VSV RESTV) = 12 nM, IC50 (EBOV) = 2.1 nM, IC50 (VSV SUDV) = 5 nM, IC50 (VSV BDBV) = 8 nM	[73]
FVM09-MR72	protein engineering		GC, 283GEWAF289, RBS	IC50 (VSV EBOV GP) = 4.7 nM, IC50 (VSV SUDV) = 10 nM, IC50 (VSV TAFV) = 2.9 nM, IC50 (VSV BDBV) = 1.1 nM, IC50 (VSV RESTV) = 6 nM, IC50 (EBOV) = 3.6 nM, IC50 (VSV SUDV) = 1.8 nM, IC50 (VSV BDBV) = 1 nM	
ADI-16061	sorting	human	HR2	IC50 (VSV EBOV GP) = 0.2 nM, IC50 (VSV TAFV) = 4.8 nM, IC50 (VSV BDBV) = 0.6 nM, IC50 (EBOV) = 0.1 nM, IC50 (VSV SUDV) = 300 nM, IC50 (VSV BDBV) = 0.3 nM	[58]
ADI-15975	sorting	human	HR2	IC50 (VSV EBOV GP) = 5.9 nM, IC50 (VSV SUDV) = 40 nM, IC50 (VSV TAFV) = 20 nM, IC50 (VSV BDBV) = 60 nM, IC50 (VSV RESTV) = 500 nM	
ADI-15750	sorting	human	GC	IC50 (VSV EBOV GP) = 9 nM, IC50 (VSV SUDV) = 34 nM, IC50 (VSV TAFV) = 0.8 nM, IC50 (VSV RESTV) = 22 nM, IC50 (EBOV) = 5.2 nM	
ADI-15968	sorting	human	GC	IC50 (VSV EBOV GP) = 12 nM, IC50 (VSV SUDV) = 43 nM, IC50 (VSV TAFV) = 4.1 nM, IC50 (VSV BDBV) = 120 nM	
ADI-15742	sorting	human	GP1–GP2 interface	IC50 (VSV EBOV GP) = 2.0 nM, IC50 (VSV SUDV) = 0.5 nM, IC50 (VSV TAFV) = 1.5 nM, IC50 (VSV BDBV) = 1.0 nM, IC50 (VSV	

				RESTV) = 0.2 nM, IC50 (EBOV) = 0.7 nM, IC50 (VSV SUDV) = 0.2 nM, IC50 (VSV BDBV) = 0.6 nM	
ADI-15878	sorting	human	GP1–GP2 interface	IC50 (VSV EBOV GP) = 0.5 nM, IC50 (VSV SUDV) = 0.3 nM, IC50 (VSV TAFV) = 1.0 nM, IC50 (VSV BDBV) = 0.5 nM, IC50 (VSV RESTV) = 0.2 nM, IC50 (EBOV) = 0.2 nM, IC50 (VSV SUDV) = 0.2 nM, IC50 (VSV BDBV) = 0.6 nM	
ADI-15881	sorting	human	GP1–GP2 interface	IC50 (VSV EBOV GP) = 30 nM, IC50 (VSV SUDV) = 60 nM, IC50 (VSV BDBV) = 70 nM	
ADI-15887	sorting	human	GP1–GP2 interface	IC50 (VSV EBOV GP) = 9 nM, IC50 (VSV SUDV) = 230 nM, IC50 (VSV BDBV) = 12 nM, IC50 (VSV RESTV) = 230 nM	
ADI-15946	sorting	human	GP1–GP2 interface	IC50 (VSV EBOV GP) = 2.5 nM, IC50 (VSV SUDV) = 40 nM, IC50 (VSV TAFV) = 8 nM, IC50 (VSV BDBV) = 0.4 nM, IC50 (EBOV) = 0.6 nM, IC50 (VSV SUDV) = 120 nM, IC50 (VSV BDBV) = 1.5 nM	
ADI-16047	sorting	human	GP1–GP2 interface	IC50 (VSV EBOV GP) = 9 nM, IC50 (VSV SUDV) = 150 nM, IC50 (VSV TAFV) = 500 nM, IC50 (VSV BDBV) = 160 nM, IC50 (VSV RESTV) = 90 nM	

Table S2. Passive immunization against EBOV

Drug	Source	Infectious dose	Model	Time after infection	Drug dose	Number of doses	Survival experimental group	Survival control group	Ref.
Serum	Convalescent human	unknown	human	3 days	450 ml, titer 1/128-1/256	2 (3, 6 A day), in combination with interferon	1/1 (100%)	Not applicable	[11]
Serum	Convalescent human	unknown	human	4–15 days	150–450 ml	1, in combination with maintenance therapy	7/8 (87,5%)	Not applicable	[12]
Plasma	Convalescent human	unknown	human	48 hr after confirmation of infection	200–250 ml	2 injections with a 15-min interval	58/84 (69%),	62%	[14]
Immunoglobulin preparation	Immune horse	30 LD_{50}	hamadryas baboon	2 hr before infection	6 ml (1/8192 titer)	Single dose	2/2 (100%)	0%	[16]
				During infection	6 ml (1/4096 titer)	Single dose	2/3 (66%)		
Immunoglobulin preparation	Immune horse	10–29 LD_{50}	hamadryas baboon	2 hr before infection	6 ml (1/8192 titer)	Single dose	2/2 (100%)	0%	[17]
				5–15 min after infection	6 ml (1/4096 titer)	Single dose	3/6 (50%)	0%	
					6 ml (1/65536 titer)	Single dose	10/10 (100%)	0%	
				30 min after infection	6 ml (1/4096 titer)	Single dose	0/5 (0%)	0%	
					6 ml (1/65536 titer)	Single dose	11/14 (80%)	0%	
				60 min after infection	6 ml (1/4096 titer)	Single dose	1/5 (20%)	0%	
					6 ml (1/16384 titer)	Single dose	5/5 (100%)	0%	
				120 min after infection	6 ml (1/4096 titer)	Single dose	0/3 (0%)	0%	
					6 ml	Single dose	2/7 (29%)	0%	

					(1/16384 titer)				
Immunoglobulin preparation	Immune horse	1,000 pfu	cynomolgus monkey	Immediately after infection	6 ml ($PRNT_{80}$ 1/2560)	Single dose	0/6 (0%)	0%	[18]
Immunoglobulin preparation	Immune horse	10000 pfu	guinea pig	Immediately after infection	1 mg/kg	Single dose	10/10 (100%)	0%	[19]
				Immediately after infection and after 3 days	1 mg/kg	Double dose	10/10 (100%)		
				After 4 days	1 mg/kg	Single dose	0/5 (0%)		
		30 LD_{50}	mouse	After 20 min	3 mg/kg	Single dose	5/20 (25%)	0%	
				After 20 min	0.3 mg/kg	Single dose	0/20 (0%)		
				After 20 min	0.03 mg/kg	Single dose	0/20 (0%)		
		1,000 pfu	cynomolgus monkey	Immediately after infection	6 ml ($PRNT_{80}$ 1/2560)	Single dose	0/6 (0%)	0%	
				Immediately after infection and after 5 days	6 ml ($PRNT_{80}$ 1/2560)	Double dose	1/3 (33%)		
				Two days before infection	6 ml ($PRNT_{80}$ 1/2560)	Single dose	0/3 (0%)		
Immunoglobulin preparation	Immune horse	1,000 LD_{50}	mouse	1 day after infection	200 µg/mouse (EC50 1.07 µg/ml)	Single dose	5/8 (62,5%)	0%	[21]
				2 days after infection	200 µg/mouse (EC50 1.07 µg/ml)	Single dose	3/8 (37,5%)		
				30 min after infection	2 mg/mouse (EC50 1,07 µg/ml)	Single dose	10/10 (100%)		
				1 day after infection	2 mg/mouse (EC50 1.07 µg/ml)	Single dose	10/10 (100%)		
				2 days after infection	2 mg/mouse (EC50 1.07 µg/ml)	Single dose	9/10 (90%)		

		1,000 LD_{50}	guinea pig	1 day after infection	20 mg/pig (EC50 1.07 µg/ml)	Single dose	6/6 (100%)	0%	
				2 days after infection	20 mg/pig (EC50 1.07 µg/ml)	Single dose	6/6 (100%)		
$F(ab')_2$	Immune horse	1,000 LD_{50}	mouse	One day after infection	200 µg/mouse (EC50 2.12 µg/ml)	Twice a day for 3 days	5/8 (62.5%)	0%	
				Two days after infection	200 µg/mouse (EC50 2.12 µg/ml)	Twice a day for 3 days	0/8 (0%)		
				30 min after infection	1 mg/mouse (EC50 2.12 µg/ml)	Twice a day for 3 days	4/9 (44%)		
				30 min after infection	2 mg/mouse (EC50 2.12 µg/ml)	Twice a day for 3 days	4/10 (40%)		
				1 day after infection	1 mg/mouse (EC50 2.12 µg/ml)	Twice a day for 3 days	8/8 (100%)		
				1 day after infection	2 mg/mouse (EC50 2.12 µg/ml)	Twice a day for 3 days	8/8 (100%)		
		1,000 LD_{50}	guinea pig	1 day after infection	20 mg/pig (EC50 1.07 µg/ml)	Twice a day for 3 days	6/6 (100%)		
				2 days after infection	20 mg/pig (EC50 1.07 µg/ml)	Twice a day for 3 days	6/6 (100%)		
Immunoglobulin preparation	Immune horse	1,000 pfu	African green monkey	1 hr after infection	20 ml (ELISA ~1:50,000; $PRNT_{50}$ 1:64)	Three times (1, 3, 10)	0/3 (0%)	0%	[20]
			cynomolgus monkey	1 day after infection	20 ml (ELISA ~1:50,000; $PRNT_{50}$ 1:64)	Once daily for 5 days, additional dose of 5 ml in two animals for 13 days	3/3 (100%)	0%	
Immunoglobulin preparation	Immune sheep	640 TCID50	guinea pig	6 hr after infection	0.5 ml (25 mg), GMT 1/11585	Nine times (1, 2, 3, 4, 5, 6, 8, 10, 12)	6/6 (100%)	17%	[23]
				48 hr after infection	0.5 ml (25 mg), GMT 1/11585	Eight times (2, 3, 4, 5, 6, 8, 10, 12)	6/6 (100%)	0%	
				72 hr after infection	0.5 ml (25 mg), GMT 1/11585	Seven times (3, 4, 5, 6, 8, 10, 12)	3/4 (75%)	0%	

Immunoglobulin preparation	Immune sheep	100 TCID50	guinea pig	3 days after infection	0.5 ml (72 mg),	Single dose	2/6 (33%)	0%	[24]
				3 days after infection	0.5 ml (24 mg),	Twice a day, six times (4, 5, 6, 8, 10, 12)	5/6 (83%)		
				4 days after infection	0.5 ml (24 mg),	Twice a day, six times (5, 6, 7, 8, 10, 12)	3/6 (50%)		
				5 days after infection	0.5 ml (24 mg),	Twice a day,five times (6, 7, 8, 10, 12)	2/6 (33%)		
Immunoglobulin preparation	Immune sheep	1,000 pfu	cynomolgus monkey	1 day after infection	6 ml (340 mg)	Eight times (1, 2, 3, 4, 5, 7, 9, 11)	4/4 (100%)	0%	[25]
				2 days after infection	6 ml (340 mg)	Eight times (2, 3, 4, 5, 6, 8, 10, 12)	2/4 (50%)		
				3 days after infection	6 ml (340 mg)	Eight times (3, 4, 5, 6, 7, 9, 11, 13)	1/4 (25%)		
Immunoglobulin preparation	Immune transchromosomal cattle	100 pfu	mouse	1 day after infection	100 mg/kg	Single dose	9/10 (90%)	10%	[26]
				2 days after infection	100 mg/kg	Single dose	0/10 (0%)		
Immunoglobulin preparation	Immune primate	1,000 pfu	rhesus monkey	2 days after infection	80 mg/kg	Triple dose (2, 4, 8)	3/3 (100%)	0%	[32]
Blood	Immune primate	1,000 pfu	rhesus monkey	Immediately after infection	6 mg/kg (ELISA titer of 1/100000)	Double dose (1, 3/4)	0/4 (0%)	0%	[29]
Serum	Immune primate (EBOV)	1,000 pfu (EBOV)	rhesus monkey	3 days after infection	11 mg/kg	Triple dose (3, 6, 9)	0/4 (0%)	0%	[31]
	Immune primate (SEBOV)	1,000 pfu (EBOV)	rhesus monkey	3 days after infection	11 mg/kg	Triple dose (3, 6, 9)	1/3 (33%)		
Monoclonal antibody 13F6-1-2	Immune mouse	10 pfu	mouse	1 before infection	100 μg	Single dose	10/10 (100%)	0%	[42]
				1 day after infection	100 μg	Single dose	10/10 (100%)		

				2 days after infection	100 µg	Single dose	3/10 (30%)		
Monoclonal antibody 6D8-1-2	Immune mouse	10 pfu	mouse	1 day before infection	100 µg	Single dose	10/10 (100%)		
				1 day after infection	100 µg	Single dose	10/10 (100%)		
				2 days after infection	100 µg	Single dose	6/10 (60%)		
Monoclonal antibody 13C6-1-1	Immune mouse	10 pfu	mouse	1 day before infection	100 µg	Single dose	10/10 (100%)		
				1 day after infection	100 µg	Single dose	10/10 (100%)		
				2 days after infection	100 µg	Single dose	8/10 (80%)		
Monoclonal antibody 133/3.16	Immune mouse	300 LD_{50}	mouse	1 day before infection	100 µg	Single dose	7/8 (87.5%)	0%	[47]
				4 days after infection	100 µg	Single dose	0/8 (80%)		
		1,000 LD_{50}	guinea pig	1 day before infection	5 mg	Double dose (-1, 2)	1/3 (33%)	0%	
				1 day before infection	5 mg	Single dose	2/3 (67%)		
				2 days after infection	5 mg	Single dose	1/3 (33%)		
Monoclonal antibody 226/8.1	Immune mouse	300 LD_{50}	mouse	1 day before infection	100 µg	Single dose	6/8 (75%)	0%	[47]
				4 days after infection	100 µg	Single dose	1/7 (14%)		
		1,000 LD_{50}	guinea pig	1 day before infection	5 mg	Double dose (-1, 2)	0/3 (0%)	0%	
				1 day before infection	5 mg	Single dose	0/3 (0%)		

				2 days after infection	5 mg	Single dose	0/3 (0%)		
Combination of chimeric antibodies ch133 and ch226	Chimeric antibodies	1,000 pfu	rhesus monkey	1 day before infection	25 mg each	Three doses (-1, 1, 3)	1/3 (33%)	0%	[48]
Monoclonal antibody KZ52	Convalescent human	10,000 pfu	guinea pig	1 hr before infection	25 mg/kg	Single dose	5/5 (100%)	0%	[37]
				1 hr after infection	25 mg/kg	Single dose	4/5 (100%)		
		1,000 pfu	rhesus monkey	One day before infection	50 mg/kg	Double dose (-1, 4)	0/4 (0%)	0%	[39]
Monoclonal antibody BDBV223	Convalescent human	1,000 pfu	guinea pig	One day after infection	5 mg	Single dose	1/5 (20%)	0%	[84]
				One day after infection	5 mg	Double dose (1, 3)	1/5 (20%)		
		1,000 pfu	mouse	1 day after infection	100 μg	Single dose	5/5 (100%)	0%	
				3 days after infection	100 μg	Single dose	0/5 (0%)		
Monoclonal antibody BDBV289		1,000 pfu	guinea pig	1 day after infection	5 mg	Single dose	3/5 (60%)	0%	
				1 day after infection	5 mg	Double dose (1, 3)	5/5 (100%)		
		1,000 pfu	mouse	1 day after infection	100 μg	Single dose	0/5 (0%)	0%	
				3 days after infection	100 μg	Single dose	0/5 (0%)		
Combination of monoclonal antibodies		1,000 pfu	guinea pig	1 day after infection	5 mg	Single dose	5/5 (100%)	0%	
				1 day after infection	5 mg	Double dose (1, 3)	5/5 (100%)		

BDBV223 and BDBV289									
Monoclonal antibody FVM04	Immune monkey	1,000 pfu	mouse	2 hr after infection	25 mg/kg	Double dose (0, 3)	15/15 (100%)	0%	[78]
Monoclonal antibody FVM04				3 days after infection	30 mg/kg	Single dose	4/10 (40%)		
Monoclonal antibody FVM02p				2 hr after infection	25 mg/kg	Double dose (0, 3)	8/15 (53%)		
Combination of monoclonal antibodies FVM02p and FVM09				2 hr after infection	15 mg/kg	Double dose (0, 3)	10/10 (100%)		
Monoclonal antibody FVM04	Immune monkey	1,000 pfu of EBOV	guinea pig	1 day after infection	5 mg	Single dose	2/6 (33%)	0%	[79]
Monoclonal antibody FVM04	Immune monkey	1,000 pfu of SUDV	guinea pig	1 day after infection	5 mg	Single dose	6/6 (100%)	0%	
Combination of monoclonal antibodies FVM04, c13C6, and c2G4	Immune monkey and Immune mouse	1,000 pfu of EBOV	guinea pig	3 days after infection	1.6 mg each	Single dose	4/6 (67%)	0%	
Combination of	Immune monkey	1,000 pfu of SUDV	guinea pig	3 days after infection	1.6 mg each	Single dose	5/6 (80%)	0%	

monoclonal antibodies FVM04, c13C6, and c2G4	and Immune mouse								
Monoclonal antibody CA45	Immune monkey	1,000 LD_{50} of EBOV	guinea pig	3 days after infection	5 mg	Single dose	3/6 (50%)	0%	[81]
Monoclonal antibody CA45	Immune monkey	1,000 LD_{50} of SUDV	guinea pig	3 days after infection	5 mg	Single dose	6/6 (100%)	0%	
Combination of monoclonal antibodies CA45 and FVM04	Immune monkey	1,000 LD_{50} of EBOV	guinea pig	3 days after infection	2.5 mg each	Single dose	6/6 (100%)	0%	
Combination of monoclonal antibodies CA45 and FVM04	Immune monkey	253 TCID50 BDBV	ferret	3 days after infection	20 mg each	Double dose (3, 6)	4/4 (100%)	0%	
Monoclonal antibody m8C4	Immune mouse	1,000 pfu	Mouse	2 hr after infection	25 mg/kg	Double dose (0, 3)	7/15 (47%)	0%	[77]
Combination of monoclonal antibodies m8C4 and 16G8				3 days after infection	15 mg/kg	Single dose	3/10 (30%)	0%	
Combination of				2 hr after infection	15 mg/kg	Double dose (0, 3)	8/10 (80%)	0%	

monoclonal antibodies m8C4 and 16G8									
Monoclonal antibody ADI-15956	Convalescent human	100 pfu	mouse	2 days after infection	100 µg	Single dose	7/10 (70%)	20%	[57]
Monoclonal antibody ADI-15974		100 pfu	mouse	2 days after infection	100 µg	Single dose	8/10 (80%)		
Monoclonal antibody ADI-15999		100 pfu	mouse	2 days after infection	100 µg	Single dose	10/10 (100%)		
Monoclonal antibody ADI-15848		100 pfu	mouse	2 days after infection	100 µg	Single dose	7/10 (70%)		
Monoclonal antibody ADI-15959		100 pfu	mouse	2 days after infection	100 µg	Single dose	5/10 (50%)		
Monoclonal antibody ADI-15878		100 pfu	mouse	2 days after infection	100 µg	Single dose	8/10 (80%)		
Monoclonal antibody ADI-15748		100 pfu	mouse	2 days after infection	100 µg	Single dose	8/10 (80%)		
Monoclonal antibody ADI-15742		100 pfu	mouse	2 days after infection	100 µg	Single dose	10/10 (100%)		
Monoclonal antibody ADI-15731		100 pfu	mouse	2 days after infection	100 µg	Single dose	4/10 (40%)		
Monoclonal antibody ADI-15744		100 pfu	mouse	2 days after infection	100 µg	Single dose	4/10 (40%)		

Monoclonal antibody ADI-16044		100 pfu	mouse	2 days after infection	100 µg	Single dose	2/10 (20%)		
Monoclonal antibody 6D6	Immune mouse	1,000 pfu	mouse	1 day after infection	100 µg	Single dose	8/8 (100%)	0%	[83]
Combination of monoclonal antibodies mAb100 and mAb114	Convalescent human	1,000 pfu	rhesus monkey	1 day after infection	50 mg/kg (4 mg/kg mAb100 and 46 mg/kg mAb114)	Triple dose (1, 2, 3)	3/3 (100%)	0%	[63]
Monoclonal antibody mAb114	Convalescent human	1,000 pfu	rhesus monkey	1 day after infection	50 mg/kg	Triple dose (1, 2, 3)	3/3 (100%)		
Monoclonal antibody mAb114	Convalescent human	1,000 pfu	rhesus monkey	5 day after infection	50 mg/kg	Triple dose (5, 6, 7)	3/3 (100%)		
MIL77, a combination of humanized c13C6, c2G4, and 4G7	Immune mouse	1,000 LD_{50}	guinea pig	3 days after infection	5 mg of the drug per animal	Single dose	5/6 (83%)	0%	[105]
					2.5 mg of the drug per animal	Single dose	4/6 (67%)		
Monoclonal antibody Q206	monkey	1,000 LD_{50}	mouse	2 days after infection	100 µg per mouse	Single dose	4/6 (67%)	0%	[60]
Monoclonal antibody Q314	monkey	1,000 LD_{50}	mouse	2 days after infection	100 µg per mouse	Single dose	2/6 (33%)	0%	
Monoclonal antibody Q411	monkey	1,000 LD_{50}	mouse	2 days after infection	100 µg per mouse	Single dose	1/6 (17%)	0%	
Combination of	human	1,025 pfu	rhesus monkey	3 and 6 days after infection	30 mg/kg	Double dose	5/5 (100%)	0%	[61,62]

monoclonal antibodies rEBOV-520 and rEBOV-548									
ADI-15878	human	100 pfu EBOV	mouse	2 days after infection	300 µg per mouse	Single dose	8/10 (80%)	5%	[58]
ADI-15878	human	1,000 pfu of SUDV	mouse	1 day after infection	300 µg per mouse	Double dose (1, 4)	20/20 (100%)	60%	
ADI-15878	human	100 pfu of BDBV	ferret	3 days after infection	300 µg per mouse	Double dose (3, 6)	3/4 (75%)	0%	
scKZ52-F4HCC	protein engineering	1,000 pfu of EBOV	mouse	1 day after infection	200 µg per mouse	Single dose	10/10 (100%)	0%	[56]
scKZ52-F4HCC	protein engineering	1,000 pfu of SUDV	mouse	1 day after infection	500 µg per mouse	Double dose (1, 4)	9/9 (100%)	20%	
FVM09-MR72	protein engineering	100 pfu of EBOV	mouse	2 days after infection	400 µg per mouse	Single dose	7/10 (70%)	10%	[73]
FVM09-MR72	protein engineering	100 pfu of SUDV	mouse	1 day after infection	400 µg per mouse	Double dose (1, 4)	10/10 (100%)	20%	

Table S3. Anti-EBOV drugs based on monoclonal antibodies

Drug	Production method	Infectious dose	Model	Time after virus infection	Drug dose	Number of doses	Survival experimental group	Survival control group	Clinical study	Ref.
ZMab, 1H3, 2G4, and 4G7	Plant cells and CHO cells	1,000 pfu (IM)	cynomolgus monkey	24 hr	25 mg/kg, IV	3 (1, 4, 7)	4/4 (100%)	0%	No (experimental drug)	[94]
		1,000 pfu (IM)	cynomolgus monkey	48 hr	25 mg/kg, IV	3 (2, 5, 8)	2/4 (50%)			[94]
		1,000 pfu (IM)	cynomolgus monkey	72 hr	50 mg/kg, IV, in combination with 1×109 pfu of rAd-IFNa, IM	3 (3, 6, 9)	3/4 (75%)	0%		[97]
		1,000 pfu (IM)	rhesus monkey	72 hr	50 mg/kg, IV, in combination with 1×109 pfu of rAd-IFNa, IM	3 (3, 6, 9)	4/4 (100%)	0%		[97]
		1,000 pfu (IM)	cynomolgus monkey	24 hr (rAd-IFNa) and 96 hr (ZMab)	50 mg/kg, IV, in combination with 1×10^9 pfu of rAd-IFNa, IM	3 (each 72 hr)	2/4 (50%)	0%		[97]
MB-003,13F6, 6D8, and 13C6	Plant cells and CHO cells	100 pfu (IM)	rhesus monkey	1 hr	CHO 50 mg/kg, IV	3 (1, 4, and 8)	2/2 (100%)	0%	No (experimental drug)	[91]
		1000 pfu (IM)	rhesus monkey	1 hr	CHO 50 mg/kg, IV, Plant 16.7 mg/kg, IV	3 (1, 4, and 8)	4/5 (80%)	100%		[91]
		1000 pfu (IM)	rhesus monkey	24 hr	Plant 16.7 mg/kg, IV	4 (1, 5, 8 and 10)	2/3 (67%)	0%		[91]
		1000 pfu (IM)	rhesus monkey	48 hr	Plant 16.7 mg/kg, IV	4 (2, 6, 8 and 10)	2/3 (67%)	0%		[91]
		1,067 pfu (IM)	rhesus monkey	103–120 hr	50 mg/kg, IV	3 (each 72 hr)	3/7 (43%)	0%		[93]
		1,000 pfu	rhesus monkey	24 hr	50 mg/kg, IV	1 (1, 4, 7)	1/3 (33%)	0%		[98]

		690 pfu	rhesus monkey	24 hr and 48 hr	50 mg/kg, IV	4 (1,5,8,10 or 2,6,8,10)	3/5 (60%)	0%		[92]
ZMapp, 2G4, 4G7, and 13C6	Plant cells and CHO cells	628 pfu	rhesus monkey	72 hr	50 mg/kg, IV	3 (3, 6, 9)	6/6 (100%)	0%		[98]
		628 pfu	rhesus monkey	96 hr	50 mg/kg, IV	3 (4, 7, 9)	6/6 (100%)	0%		[98]
		628 pfu	rhesus monkey	120	50 mg/kg, IV	3 (5, 8, 11)	6/6 (100%)	0%		[98]
		unknown	human	4–7 days after manifestations of symptoms	50 mg/kg, IV	3 (three-day interval)	28/36 (78%)	63%	NCT02342171 Randomized, controlled, multicenter Phase 1/2 trial	[100]
		unknown	human	1 day after manifestations of symptoms	50 mg/kg, IV	3 (three-day interval)	85/169 (50,3%)	Not applicable	Phase II/III Completed the Democratic Republic of the Congo NCT03719586, PALM (failed drug)	[105]
MIL77	CHO cells	1,000 pfu	rhesus monkey	3 days after infection	50 mg/kg, IV	3 (3, 6, 9)	3/3 (100%)	0%		[104]
		unknown	human	unknown	unknown	unknown	unknown	unknown	Phase I clinical trial (failed drug)	[105]
Ebanga, mAb 114	CHO cells	1,000 pfu	rhesus monkey	1,2,3 days after infection	50 mg/kg, IV<br>50 mg/kg, IV	3<br>1	3/3 (100%)<br>3/3 (100%)	0%	Phase I Completed USA	[63,65]

				5 days after infection					NCT03478891,	
		unknown	human	unknown, virus infection is confirmed by RT-PCR	50 mg/kg, IV	1	113/174 (64,9%)	Not applicable	Phase II/III Completed Congo, USA PALM, NCT03719586 (approved drug)	
Inmazeb (REGN-EB3) REGN3470, REGN3479, REGN3471	low fucose CHO cells	1,000 pfu	rhesus monkey	5,8,11 days after infection	50 mg/kg, IV	3	18/18 (100%)	0%	Phase I Completed USA NCT02777151	[68,109]
		unknown	human	unknown, virus infection is confirmed by RT-PCR	150 mg/kg, IV	1	103/155 (33,5%)	Not applicable	Phase II/III Completed the Democratic Republic of the Congo NCT03719586, PALM (approved drug)	
